# Bio-based Films from Linter Cellulose and Its Acetates: Formation and Properties 

**DOI:** 10.3390/ma6062410

**Published:** 2013-06-14

**Authors:** Daniella L. Morgado, Bruno V. M. Rodrigues, Erika V. R. Almeida, Omar A. El Seoud, Elisabete Frollini

**Affiliations:** 1 Macromolecular Materials and Lignocellulosic Fibers Group, Center for Research on Science and Technology of BioResources, Institute of Chemistry of São Carlos, University of São Paulo, P.O. Box 780, São Carlos 13560-970, Brazil; E-Mails: danilury@gmail.com (D.L.M.); bruno.manzolli@gmail.com (B.V.M.R.); emaildaerikinha@yahoo.com.br (E.V.R.A.); 2 Institute of Chemistry, University of São Paulo, P.O. Box 26077, São Paulo 05513-970, Brazil

**Keywords:** bio-based films, linter cellulose, cellulose acetates, LiCl/DMAc solvent system

## Abstract

This paper describes the results obtained on the preparation of films composed of linter cellulose and the corresponding acetates. The acetylation was carried out in the LiCl/DMAc solvent system. Films were prepared from a LiCl/DMAc solution of cellulose acetates (degree of substitution, DS 0.8–2.9) mixed with linter cellulose (5, 10 and 15 wt %). Detailed characterization of the films revealed the following: (i) they exhibited fibrous structures on their surfaces. The strong tendency of the linter cellulose chains to aggregate in LiCl/DMAc suggests that these fibrous elements consist of cellulose chains, as can be deduced from SEM images of the film of cellulose proper; (ii) the cellulose acetate films obtained from samples with DS 2.1 and 2.9 exhibited microspheres on the surface, whose formation seems to be favored for acetates with higher DS; (iii) AFM analysis showed that, in general, the presence of cellulose increased both the asperity thickness and the surface roughness of the analyzed films, indicating that cellulose chains are at least partially organized in domains and not molecularly dispersed between acetate chains; and (iv) the films prepared from cellulose and acetates exhibited lower hygroscopicity than the acetate films, also suggesting that the cellulose chains are organized into domains, probably due to strong intermolecular interactions. The linter and sisal acetates (the latter from a prior study), and their respective films, were prepared using the same processes; however, the two sets of films presented more differences (as in humidity absorption, optical, and tensile properties) than similarities (as in some morphological aspects), most likely due to the different properties of the starting materials. Potential applications of the films prepared in tissue engineering scaffold coatings and/or drug delivery are mentioned.

## 1. Introduction

In recent decades, the world has followed a global appeal to protect the environment by reducing the emission of greenhouse gases, by saving fossil resources, and by increasing the development of bio-based products. In this scenario, lignocellulosic and cellulosic fibers emerged as one of the most important raw materials to be considered. 

Lignocellulosic fibers can be employed as, e.g., a reinforcing agent in polymeric matrices [1,2,3], whereas their main components, namely lignin, hemicellulose and cellulose, can be separated from each other for different applications [4,5,6]. The delignification of lignocellulosic fibers, such as wood or annual non-woody fibers, has been widely investigated because of the interest of the paper industry [7,8], and other interests such as those of biorefineries that produce cellulosic ethanol [9,10,11,12,13,14,15], or the area of materials focused on cellulose and its derivatives [16,17,18,19,20]. It is expected that the availability of celluloses from different sources, hence with different properties, will increase significantly. Raw materials such as cotton linter, which is employed in the present study, are not only important in the textile area [21,22,23,24], but can also be very attractive for other applications because there is no need to remove lignin. 

It is known that celluloses obtained from different sources have distinct physico-chemical characteristics. Focusing on the expansion of applications of cellulose, it is important to evaluate how the characteristics of the starting biopolymer influence the pathway of a certain process [25,26,27], as well as the properties of the respective final product. The interest in exploring the behavior of different celluloses under the same process motivated the study on the heterogeneous acetylation of microcrystalline and sisal celluloses [28], where very different results were found when comparing the two celluloses. In another study, the dissolution of microcrystalline cellulose, mercerized linter and mercerized sisal celluloses was explored [26]. The average degrees of polymerization (DP) of these celluloses were, respectively, 126, 377 and 544; the corresponding indices of crystallinity (Ic) were 83%, 71% and 65%. Mercerized linter and sisal presented 92% and 97% as α-cellulose content, respectively [25]. These properties, among others, played a significant role in the solubility of cellulose [26]. Whereas sisal cellulose can be directly dissolved in LiCl/DMAc, linter cellulose, which possesses longer organized domains and a smaller pore volume than sisal, can only be completely dissolved after some pre-treatment, in particular, mercerization. This treatment has resulted in reduction of both Ic and the crystallite size, as well as a higher homogeneous pore size distribution [25,27]. 

The influence of the physico-chemical characteristics of the starting cellulose goes beyond the step of dissolution: it also influences the processes carried out with these solutions, such as derivatization and/or the film formation. Thus a previous study on the homogeneous acetylation (LiCl/DMAc as the solvent system) of microcrystalline, mercerized linter and mercerized sisal celluloses has shown that the reactivity followed the order microcrystalline > mercerized sisal > mercerized linter. The accessibility of cellulose during acetylation (LiCl/DMAc), hence the observed degree of substitution (DS), was found to depend on DP and α-cellulose content of the starting biopolymer; this has been correlated with the aggregation state of the dissolved chains [26].

Regarding the influence of the starting cellulose on film formation, the dissolution of chitosan/sisal cellulose [29] and chitosan/linter cellulose [30] in NaOH/thiourea aqueous solutions was investigated, aiming at the preparation of films. Under the conditions employed, the dissolution process led to a decrease of approximately 40% and 80% of the DP of linter and sisal, respectively. Among the possible reasons for this significant difference, the lower crystallinity of sisal cellulose was highlighted [30]. Additionally, the film based on linter cellulose exhibited higher thermal stability, probably due to the lower α-cellulose content of sisal cellulose, as a consequence of the presence of hemicelluloses. This polysaccharide decomposes thermally at lower temperatures, when compared to cellulose [29,30].

The preceding discussion shows the importance of understanding the dependence of a certain aspect of cellulose chemistry, e.g., the efficiency during cellulose derivatization, or the properties of films obtained therefrom on the properties of the starting materials [25,26,27,28,29,30]. The present investigation is related to this theme, namely, the performance of celluloses with different characteristics in the synthesis of cellulose acetates for the preparation of films; LiCl/DMAc has been employed as the solvent system for both processes. In this manuscript, new data on linter cellulose acetates and their films are described, and compared with results obtained in a prior study, where sisal cellulose was used [17]. 

Previous studies have shown the advantages of using a homogeneous medium for the esterification of different starting celluloses [26,27]. Although ionic liquids are considered attractive solvents for the derivatization of cellulose in a homogeneous medium [31,32,33], LiCl/DMAc has remained a popular choice, *inter alia*, because of the negligible degradation of the biopolymer during its derivatization, and the efficient recovery of pure solvent by distillation [17,26,34,35,36,37,38,39]. 

Cellulose acetates are considered the most important cellulose derivative, because of their wide range of industrial applications. These include, e.g., thickeners in cosmetics and food products; membranes for drug release, adhesives and biomolecule immobilization [40,41,42,43]. The final properties of the cellulose acetate have a direct dependence on the method of synthesis, the degree of substitution and thus the uniformity of the distribution of the acetate groups in the anhydroglucose unit and along the cellulose backbone**.**

A prior study was carried out on the acetylation of mercerized sisal cellulose in LiCl/DMAc, for further application in bio-based films. These were prepared from LiCl/DMAc solutions of neat cellulose acetates or cellulose and from mixed cellulose acetate/cellulose solutions [17]. In this prior study, the formation of chain aggregates during the dissolution of cellulose in LiCl/DMAc, which was detected in other studies [26,44], was considered a positive aspect, because of the possibility of generating supramolecular structures on the *nanoscale* directly in the process of film preparation, and the possible action of these supramolecular structures as a reinforcement of acetate-based matrices. 

In the present study, an investigation was carried out on the evaluation of the influence of another type of cellulose, mercerized linter, on the properties of bio-based films prepared from linter cellulose and their acetates. Some of the expected differences between the films prepared from sisal cellulose [17] and those investigated in the present study may arise from the considerable differences in the behavior of sisal and linter celluloses in solution [26,27], which may affect the process of film formation. Thus, bio-based films prepared from linter cellulose acetates/linter cellulose with different properties than those prepared from sisal cellulose are expected due to the distinct physical-chemical properties of these celluloses, such as DP, Ic, porosity, and also due to the different degree of aggregation of these celluloses in LiCl/DMAc [26].

Linter cellulose-based acetates with different DS were prepared in LiCl/DMAC solutions. The starting material and the respective bio-based films prepared were characterized by various techniques, such as X-ray diffraction and TGA, and the films were also characterized by SEM, AFM, and humidity absorption. Solutions prepared from acetates with different DS and/ or mixed with different proportions of cellulose were used in film preparation. Although some of the films prepared were not examined by all the (film) characterization techniques, *vide infra*, the results obtained are sufficient to evaluate the similarities and differences between the properties of these films and those prepared from sisal cellulose, as well as to detect some possible applications for the materials described here. 

## 2. Results and Discussion

### 2.1. Properties of Linter Cellulose 

The effects of alkali treatment on linter cellulose were discussed in a previous paper [44]; it led to a decrease in DP (from 408 to 358) as determined by viscometry, $\bar{DP}v$, and in Ic% (from 79% to 73%) as determined by X-ray diffraction. The alkali treatment also increased the α-cellulose content (from 91% to 95%). Only after this treatment was the complete dissolution of linter cellulose in LiCl/DMAc possible, whereas sisal cellulose dissolves after and before mercerization [25]. This mercerized linter cellulose was used to prepare the cellulose acetates.

### 2.2. Characterization of Cellulose Acetates

The DS of cellulose acetates can be calculated using **^1^**H NMR from the ratio between the areas of the hydrogens of the glucose ring (δ 2.90–5.10 ppm) and the singlet of the methyl moiety of the acetate group (δ 1.70–2.20 ppm, spectra not shown) [45,46,47,48]. 

A previous study on the acetylation of microcrystalline cellulose, MCC, mercerized linter and mercerized sisal celluloses has shown the relevance of the physical state of the dissolved bioplymer, namely, the degree of aggregation of the cellulose chains, to the derivatization efficiency. Thus the dependence of DS on the molar ratio Ac_2_O/AGU (acetic anhydride/anhydroglucose units) can be fitted by using a first-order exponential decay equation; the reaction efficiency is inversely dependent on the aggregation number of the cellulose chains (calculated from static light scattering measurements) [26].

Table 1 displays the reaction conditions and the DS of the cellulose acetates prepared in this study.

**Table 1:** Molar ratio of acetic anhydride/anhydroglucose units (Ac_2_O/AGU) used and experimental degree of substitution (DS) of cellulose acetates obtained.

Molar ratio Ac_2_O/AGU	DS	Sample code
1.5	0.8	Ac0.8
5.0	1.5	Ac1.5
9.0	2.1	Ac2.1
12.0	2.9	Ac2.9

The results listed in Table 1 indicate that the accessibility of the hydroxyl groups was hindered by the aggregation of the cellulose chains, and thus an excess of Ac_2_O was required to achieve the targeted DS, in agreement with previous studies [17,26].

### 2.3. Characterization of Cellulose, Cellulose Acetate and Bio-Based Cellulose Acetate/Cellulose Films

Table 2 lists the films that were prepared from the LiCl/DMAc solution of neat cellulose acetates (FAcDS) and the cellulose acetates mixed with different proportions of cellulose (FAcDSCell%). 

**Table 2:** Cellulose acetate films obtained from the LiCl/DMAc solution.

Acetate (DS)	Acetate (wt %)	Cellulose (wt %)	Sample Code
0.8	100	0	FAc0.8
	95	5	FAc0.8Cell5
	90	10	FAc0.8Cell10
	85	15	FAc0.8Cell15
1.5	100	0	FAc1.5
	95	5	FAc1.5Cell5
	90	10	FAc1.5Cell10
	85	15	FAc1.5Cell15
2.1	100	0	FAc2.1
	95	5	FAc2.1Cell5
	90	10	FAc2.1Cell10
	85	15	FAc2.1Cell15
2.9	100	0	FAc2.9
	95	5	FAc2.9Cell5
	90	10	Fac2.9Cell10
	85	15	FAc2.9Cell15

#### 2.3.1. Elemental Analysis and Atomic Absorption

Cellulose (FCell), cellulose acetates (FAcDS) and bio-based cellulose acetate/cellulose (FAcDSCell%) films were analyzed by atomic absorption and elemental analysis to verify the possible presence of residual lithium (from lithium chloride) and nitrogen (from DMAc), respectively. The starting materials used to prepare the films, cellulose and cellulose acetates, were also analyzed. 

Elemental analysis showed that, though the products were exhaustively washed with methanol, the dissolution and subsequent acetylation of cellulose in LiCl/DMAc led to cellulose acetates with a small content of lithium (the highest percentage detected was 0.7%). However, lithium was not found in the film prepared from the acetates, indicating that the washing process of the films completely eliminated the electrolyte from the solvent system. 

The contents of nitrogen in the original samples (cellulose and cellulose acetates) and their respective films were small (from 0.02% to 0.1%). It must be noted that 0.1% of N was detected in the starting cellulose, before it was dissolved in the LiCl/DMAc solvent system. This result may be a consequence of the presence of some contaminant present in the starting cellulose, carried over to the acetates and the films.

#### 2.3.2. Size-Exclusion Chromatography (SEC) of DS 0.8 acetate (Ac0.8), Mixed or not Mixed with Cellulose, and of the Film FAc0.8Cell10 (Redissolved in LiCl/DMAc)

**Figure 1:**
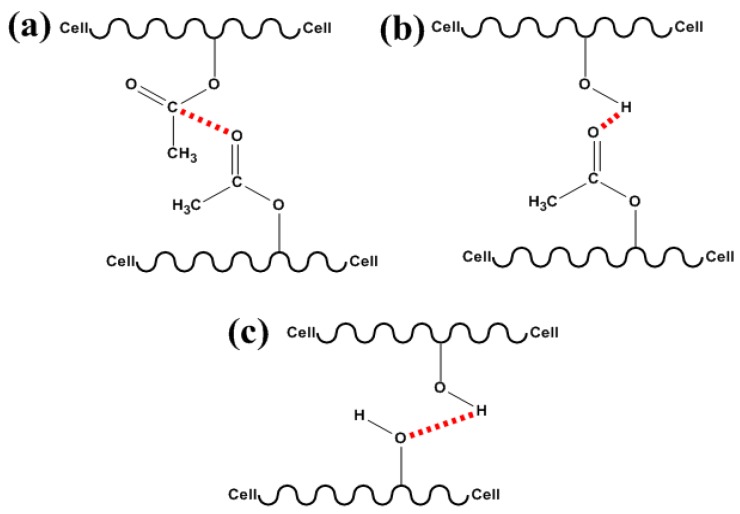
Schematic representations of possible interactions between partially substituted acetate chains (**a**–**c**) or acetate/cellulose chains (**b**,**c**) or cellulose/cellulose chains (**c**).

The aim of this chromatographic analysis was to evaluate whether it would be possible to detect interactions between acetate/acetate, acetate/cellulose, cellulose/cellulose chains (Figure 1). These interactions may induce aggregate formation, leading to an increase in the average molar mass. Therefore, low DS acetate (0.8) with a high amount of unsubstituted hydroxyl groups was chosen for this study, which included SEC analysis of the following samples (all in LiCl/DMAc): cellulose acetate Ac0.8 (mixed or not mixed with 10 wt % cellulose); and the bio-based film FAc0.8Cell10 (obtained by adding 10 wt % of cellulose to Ac0.8 acetate). 

**Figure 2:**
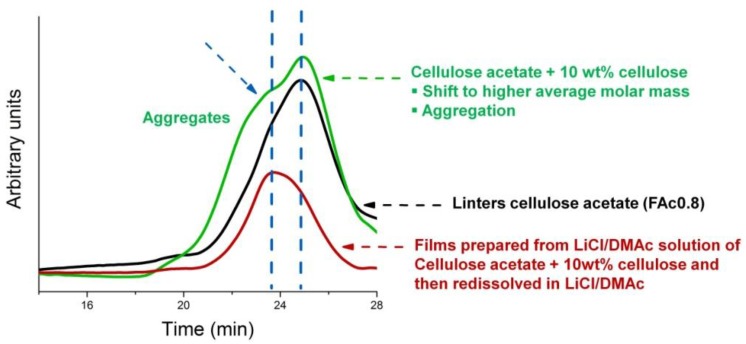
Size-Exclusion Chromatography (SEC) chromatogram obtained for Ac0.8 (mixed or not mixed with 10 wt % cellulose) and FAc0.8Cell10, both dissolved in LiCl/DMAc.

Figure 2 displays the chromatograms for Ac0.8, mixed or not mixed with 10 wt % cellulose, and those for FAc0.8Cell10. 

Figure 2 shows that the addition of 10 wt % of cellulose to the solution of the cellulose acetate Ac0.8 (before the preparation of the film, green curve) caused a significant broadening of the curve, when compared to the curve of the neat acetate (black curve). This broadening can be traced to the aggregation involving cellulose chains. In the region that suggests the presence of aggregates (see the arrow, green curve) there appeared a shoulder at approximately 23.5 min in the curve corresponding to the starting acetate Ac0.8 + 10 wt % cellulose (green curve), and a maximum in the curve corresponding to the redissolved film (FAc0.8Cell10, red curve). This result shows that the presence of cellulose chains favored the formation of aggregates both when mixed with the starting acetate (green curve) and when used to prepare the film (FAc0.8Cell10, red curve).

The development of aggregates of cellulose chains and/or of their derivatives, such as acetate chains, may also be investigated through viscosimetric measurements. The parameters used in these studies are the viscosity of the polymer solution and the Huggins constant (k_H_). In good solvents, in which the polymer chains are solvated and the polymer–solvent interactions are dominant, k_H_ reaches values up to approximately 0.40. Values of k_H_ increase (>0.55) with decrease of the solvent quality. High values for k_H_ are attributed to the presence of supramolecular structures (aggregates) in solution [49,50,51].

In a prior study [44], the solution behavior of mercerized linter and Ac0.8 in LiCl/DMAc was evaluated. In the viscometric study, the solutions were prepared at a range of very low concentrations (0.002–0.007 g mL^−1^) to suppress chain aggregation; k_H_ values were calculated for cellulose and Ac0.8 were 1.8 and 0.3, respectively [44]. These results indicated that, even at low concentration, the cellulose chains have a high tendency towards aggregation because k_H_ >> 0.55 and that this tendency is considerably higher than that of the acetate chains (k_H_ 0.3). These results suggested that the cellulose chains would tend to interact with themselves in a mixed acetate/cellulose LiCl/DMAc solution. This trend may lead to domains of cellulose chains inserted into a matrix of cellulose acetates when the solvent is eliminated, as occurred during the preparation of the films in the present study, as discussed below.

The introduction of acetate groups in the cellulose chains can lead to two opposing effects: (i) the expansion of chains due to steric repulsion between acetate groups, which, in principle, would increase the polymer/solvent interactions (LiCl/DMAc); (ii) a decrease of the polymer/solvent interactions because the replacement of hydroxyl by an acetate group decreases the interactions cel–OH - - - Cl^− ^(from LiCl), which are important in the dissolution of cellulose in LiCl/DMAc and also most likely for their derivatives, such as acetates [44]. One of these effects can outweigh the other, depending on the conditions under consideration. 

Unsubstituted OH groups predominate in Ac0.8, and the respective value of k_H_ (0.3) indicates that the solution can be considered as molecularly dispersed in the low concentration range employed. Prior studies [46,52] showed that, when using LiCl/DMAc as the solvent system, C6-OH of the AGU is acetylated first. For DS 0.8, practically only this primary hydroxyl group is substituted, which can even facilitate intramolecular interactions involving the acetate group and the surrounding hydroxyl groups, thus suppressing intermolecular interactions between acetate chains.

The results of the prior viscometric study [44] corroborated our conclusion regarding the SEC results, *i.e*., in a mixed acetate/cellulose LiCl/DMAc solution, the aggregation of cellulose chains may be favored compared to the aggregation of the acetate chains.

In the preparation and characterization of films, acetates with DS values other than 0.8 were also considered in the present study, and a cellulose film was also prepared. An attempt was made to diversify the content of cellulose in these films as well, as described below. 

#### 2.3.3. Morphological Surface Analyses

##### ***Scanning Electron Microscopy (SEM)*** 

**Figure 3:**
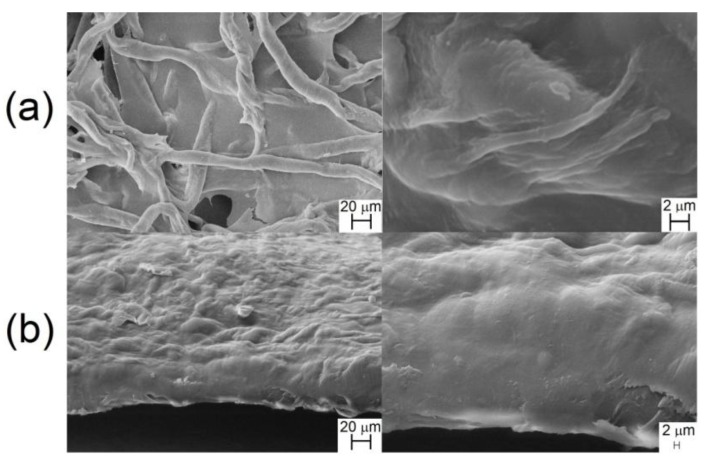
Scanning Electron Microscopy (SEM) micrographs of the surface (**a**) and the cross-section (**b**) of the cellulose film.

The images of the surfaces and the cross-section of the cellulose film (FCell) are shown in Figure 3.

The SEM images of the cellulose film (FCell) show fibrous structures on the surface of the film (Figure 3a), suggesting that aggregates of cellulose chains, generated in LiCl/DMAc solution during the film formation, organized themselves during the removal of the solvent and formed fibrous structures. The images of the cross-section of the FCell also show fibrous structures (Figure 3b). This finding corroborates the results of the viscometric analysis previously mentioned [44]. In the present study, the films were prepared from solutions of linter cellulose in LiCl/DMAc approximately ten times more concentrated than those used in the viscometric analysis, which most likely favored the aggregation of cellulose chains during the preparation of the films. The mercerized sisal cellulose film, exhibited a more compact and smoother surface [17] when compared to the linter cellulose film (Figure 3a). In a prior study [27], as already mentioned, it was found that linter cellulose similar to the one employed in the present study exhibited chains with a higher “aggregation state” than sisal cellulose chains in LiCl/DMAc solutions, which was mainly attributed to a higher $\bar{DP}v$ (544 for mercerized sisal and 370 for mercerized linter) and the presence of hemicellulose in the sisal pulp.

**Figure 4:**
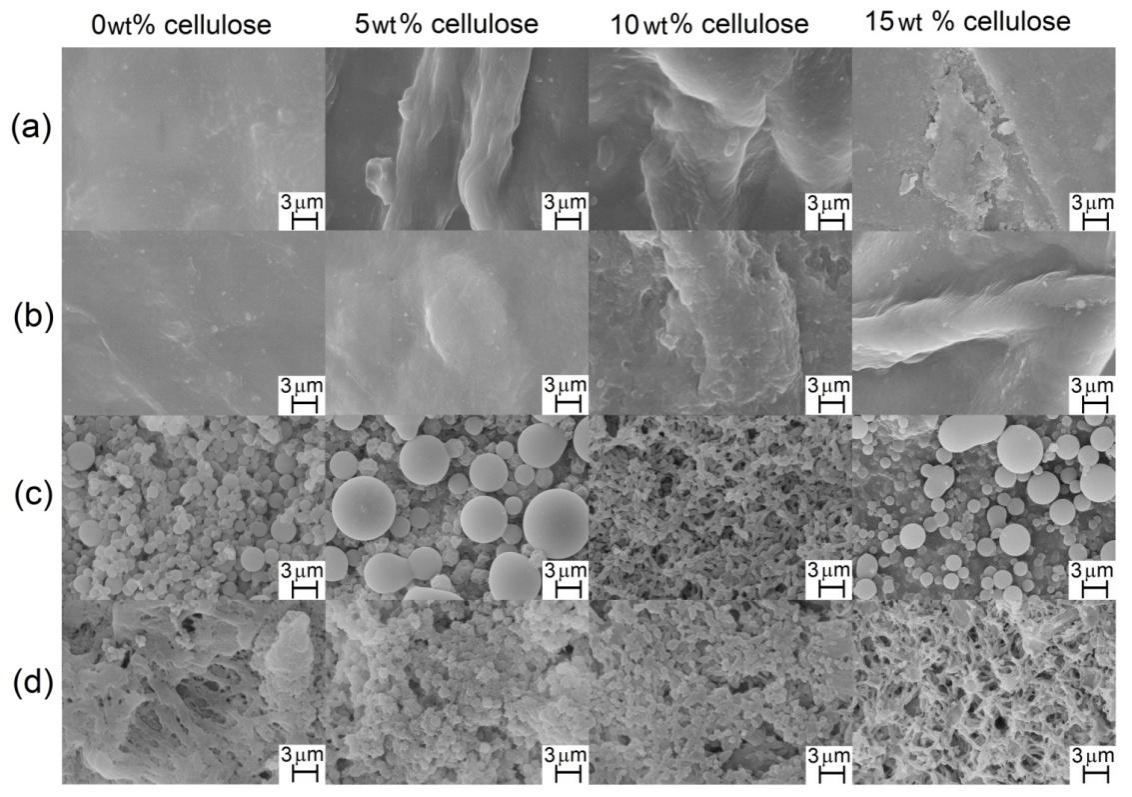
SEM micrographs of the surface view of cellulose acetate (FAcDS) and cellulose acetate/cellulose (FAcDSCell%) films with the respective percentages of cellulose: (**a**) DS 0.8; (**b**) DS 1.5; (**c**) DS 2.1 and (**d**) DS 2.9.

Figure 4 shows the SEM images of the surfaces of cellulose acetate (FAcDS) and cellulose acetate/cellulose films with (FAcDSCell%) or without cellulose (FAcDS).

The surfaces of the films obtained from the acetates with DS 0.8 and 1.5 (Figure 4a,b, respectively) were smoother than the surfaces of the other films. Acetates with low DS have a higher number of free hydroxyl groups to participate in hydrogen bonding, which may favor the homogeneity of the films. The viscometric study mentioned previously [44] showed that a low DS acetate, such as 0.8, has a low tendency to aggregate in LiCl/DMAc solutions, which can also contribute to the observed homogeneity. In the films prepared from solutions of cellulose acetates, DS 0.8 and 1.5, plus cellulose (5, 10 and 15 wt %), the presence of fibrous structures on the surfaces is noted, with thickness ranging approximately from 5 to 11 µm. The strong tendency of the linter cellulose chains to aggregate in LiCl/DMAc suggests that these fibrous elements consist of cellulose chains.

The cellulose acetate films obtained from acetates with DS 2.1 and 2.9 (Figure 4c,d, respectively) had microspheres (b) on their surface films. In the prior study on sisal cellulose acetate films [17], microspheres were also observed for the DS 2.2 acetate films. The formation of these microspherical elements seems to be favored for acetates with higher DS. 

The viscosimetric study mentioned earlier for linter cellulose and Ac0.8 also included Ac2.1 [44]. The k_H_ value obtained was 0.8, indicating that, at 25 °C, in the low concentration range solutions used in that study, the Ac2.1 acetates chains are already aggregated. This finding indicated that the introduction of a higher number of acetate groups, compared to Ac0.8, favored the aggregation. The larger volume of the acetate as compared with the hydroxyl group can expand the chains due to steric repulsion. Additionally, the lower number of free hydroxyl groups in Ac2.1 can affect the interaction with the LiCl/DMAc solvent system, as previously mentioned, favoring the interaction between acetate chains. The formation of microspheres in the films prepared from Ac2.1 and Ac2.9 may be due to the formation of acetate chain aggregates in the solutions. The formation of these aggregates was fostered in the preparation of the films because the solutions used were approximately ten times more concentrated than those of the viscosimetric study (see experimental section) [44]. These aggregates may have generated the supramolecular structures with spherical morphology (Figure 4) when the solvent was slowly eliminated during the film formation. 

Zhou and Chen [53] studied chitosan/cellulose acetate microspheres with hydrophobic core and hydrophobic coating as a potential drug delivery system. The cellulose acetate used in that study had an acetyl content equal to 55%. Different model drugs were employed in that work, and the results showed that microspheres obtained from chitosan and cellulose acetate present good effects on the controlled release of drugs with different hydrophilicities. Wang *et al*. [54] and Meier *et al*. [55] developed membranes based on acetates for drug delivery. The suitability of materials based on cellulose acetates for such applications, combined with the microspherical elements exhibited for some films in the present study, may be useful for the development of materials for controlled drug release.

**Figure 5:**
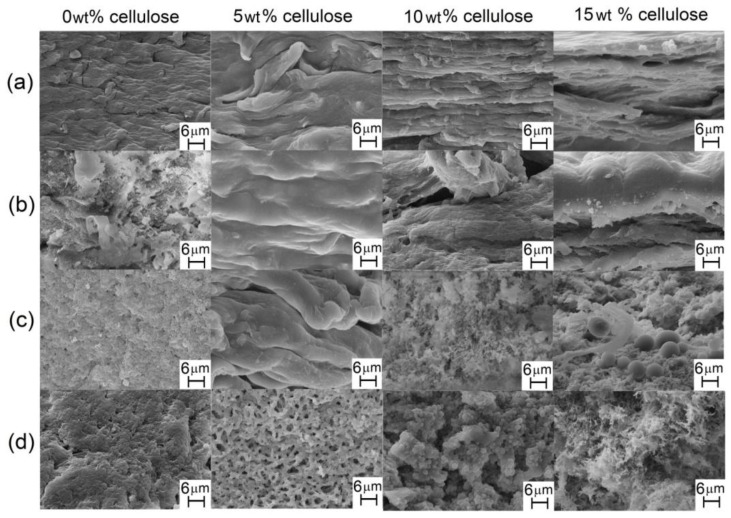
SEM micrographs of the cross-section of cellulose acetate (FAcDS) and cellulose acetate/cellulose (FAcDSCell%) films with the respective percentages of cellulose: (**a**) DS 0.8; (**b**) DS 1.5; (**c**) DS 2.1 and (**d**) DS 2.9.

Figure 5 displays the SEM images of the cross-section of the films. Figure 5 shows that most of the films exhibited a rough cross-section, although the ones obtained from Ac0.8 seem more homogeneous (Figure 5a). The cross-section of the films FAc 0.8 (Figure 5a) and FAc 1.5 (Figure 5b) exhibited microfibers that ranged approximately from 0.7 µm to 17 µm, which are a little thicker than those observed in their surfaces (Figure 4a and Figure 4b, respectively). Some images (such as those corresponding to FAc0.8Cell5, FAc0.8Cell10, FAc1.5Cell5) indicated a tendency of these fibrous elements towards organization into layers in the inner parts of the films. In general, the cross-sections of FAc2.1Cell5, FAc2.1Cell10 and FAc2.1Cell15 (Figure 5c) and of FAc2.9Cell10 and FAc2.9Cell15 (Figure 5d) exhibited a different morphology than that of others films. The microspheres are shown in some images as hollow (Figure 5d, FAc2.9Cell5) or closed (Figure 5c, FAc2.1Cell15; Figure 5d, FAc2.9Cell10) elements.

The presence of the fibrous elements and/or microspheres on a microscopic scale, in both the films prepared from cellulose and those from cellulose acetates, introduced a second phase in these materials that influenced their optical properties, leading thus to opaque films. The films with spherical elements, prepared from sisal cellulose and the respective acetates using LiCl/DMAc as the solvent system, mentioned earlier, also showed opacity. However, some of those films showed no spherical or fibrous elements and exhibited transparency [17].

##### ***Atomic Force Microscopy (AFM)*** 

**Figure 6:**
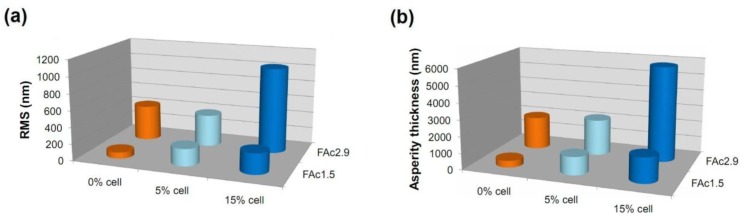
RMS surface-roughness values and asperity thickness of cellulose acetate and cellulose acetate/cellulose films, DS 1.5 and 2.9 (FAc1.5 and FAc2.9, respectively, and 0, 5 and 10 wt % of cellulose)

Only some of the films were analyzed via AFM due to the experimental difficulties in analyzing the others, which was caused by the inadequacy of the surfaces of the films for this analysis. Figure 6 shows the RMS surface-roughness values and the asperity thickness measured from the AFM images (figures not shown) of cellulose acetate (FAcDS) and bio-based cellulose acetate/cellulose (FAcDSCell%) films with DS 1.5 and 2.9.

In general, the presence of cellulose increased both the asperity thickness and the surface roughness, indicating that cellulose chains are at least partially organized in domains and not molecularly dispersed between the corresponding acetate chains. Note that the same trend was observed in films prepared from acetates with DS 1.5, which did not exhibited microspheres (Figure 4), and in those prepared from acetates with DS 2.9, which exhibited microspheres in the respective morphologies (Figure 4). The presence of microspheres in the films prepared from acetate with DS 2.9 led to higher values of RMS and asperity thickness when compared to films prepared from acetates with DS 1.5 (Figure 6).

#### 2.3.4. Crystallinity Index (Ic) 

**Figure 7:**
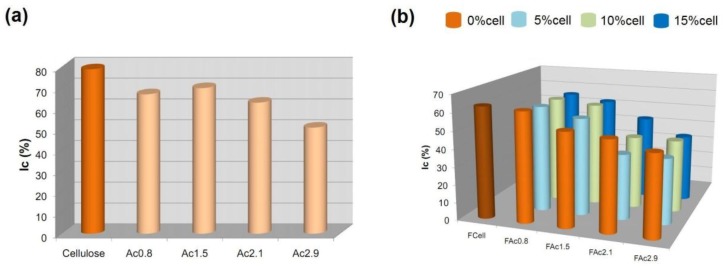
Crystallinity index (Ic) of (**a**) original cellulose and cellulose acetates and (**b**) cellulose (FCell), cellulose acetate (FAcDS) and cellulose acetate/cellulose (FAcDSCell%) films.

Figure 7 depicts the crystallinity index (Ic) obtained from X-ray diffractograms (not shown) for each film considered in this study. The original cellulose and cellulose acetates, *i.e*., the material used to prepare the films, were also analyzed. 

As shown in Figure 7, the dissolution of the starting cellulose (Ic 79%, Figure 7a) in the solvent system LiCl/DMAc and the subsequent preparation of cellulose film (FCell, Ic 63%, Figure 7b) led to a decrease in the percentage of crystalline domains. The acetylation of cellulose chains led to products with lower crystallinity because all acetates exhibited a lower I_c_ than linter cellulose, both in powder form (Figure 7a) and as films (Figure 7b).

The films prepared from cellulose acetate with DS 0.8 (FAc0.8) presented close crystallinity whether prepared with or without cellulose (Figure 7b). The crystallinity of the films obtained from acetate with DS 1.5 (FAc1.5) increased with the percentage of cellulose added to the cellulose acetate. These results suggested that domains of cellulose chains were formed, which could correspond to the fibrous structures observed in the SEM images (Figure 4b). 

The films prepared from acetates with DS 2.1 and 2.9 plus cellulose (FAc2.1 and FAc2.9, with 5, 10 and 15 wt % of cell, Figure 7b) exhibited lower crystallinity than the respective neat films (0 wt % of cell), suggesting that the introduction of cellulose chains also influences the arrangement of the chains for these acetates with higher DS. However, mainly for the set FAc2.1, comparing the cellulose-containing films (5, 10 and 15 wt %) reveals a tendency of increasing crystallinity as a function of increasing the cellulose content, in agreement with our argument that at least part of the cellulose chains are auto-organized into domains in these films (Figure 7b).

#### 2.3.5. Humidity Absorption 

**Figure 8:**
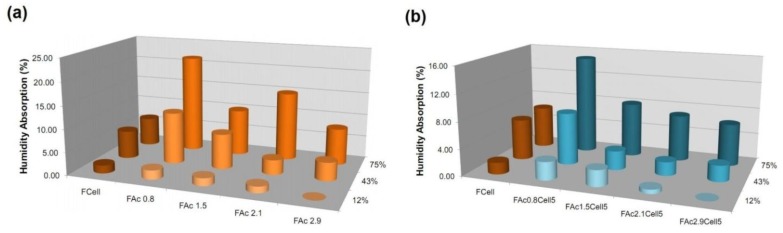
Humidity absorption of (**a**) cellulose (FCell), cellulose acetate (FAcDS) and (**b**) bio-based cellulose acetate/5 wt % of cellulose (FAcDSCell5) films as a function of different relative humidities.

The films containing lower percentages of cellulose, namely 5 wt % (FAcDSCell5), were chosen to evaluate the influence of the presence of cellulose in the films on their capacity to absorb humidity (Figure 8b). Films prepared from neat acetates were also analyzed for comparison (Figure 8a).

The cellulose film (FCell) has a higher number of free hydroxyl groups than the films prepared from acetates (FAcDS, Figure 8a) and/or the films containing cellulose (FAcDSCell5, Figure 8b). However, when compared with FAcDSCell5 films, FCell absorbed a lower, or not so much superior, amount of humidity (Figure 8). FCell has higher crystallinity than the FAcDSCell5 films (Figure 7b) and, more importantly, the crystalline domains are inserted into fibrous elements, as indicated by the SEM image (Figure 4). Thus, despite the intrinsic hydrophilicity of FCell, the interaction with water molecules was attenuated by the organization of the cellulose chains in domains where the intermolecular interactions between the chains are strong. The film prepared from sisal cellulose (previous study), which did not show fibrous elements, absorbed more moisture than the films prepared from the sisal cellulose acetates [17].

The comparison of the humidity absorption of films prepared from neat acetates (FAcDS, Figure 8a) and their films with 5% cellulose (FAcDSCell5, Figure 8b) showed that, in general, the presence of cellulose led to films with less affinity for water. This finding suggested that the cellulose chains have auto-organized into domains, where they were involved in strong intermolecular interactions, which attenuated the interactions with the water molecules, as also indicated by other results discussed previously.

#### 2.3.6. Thermogravimetric Analysis (TGA) 

**Figure 9:**
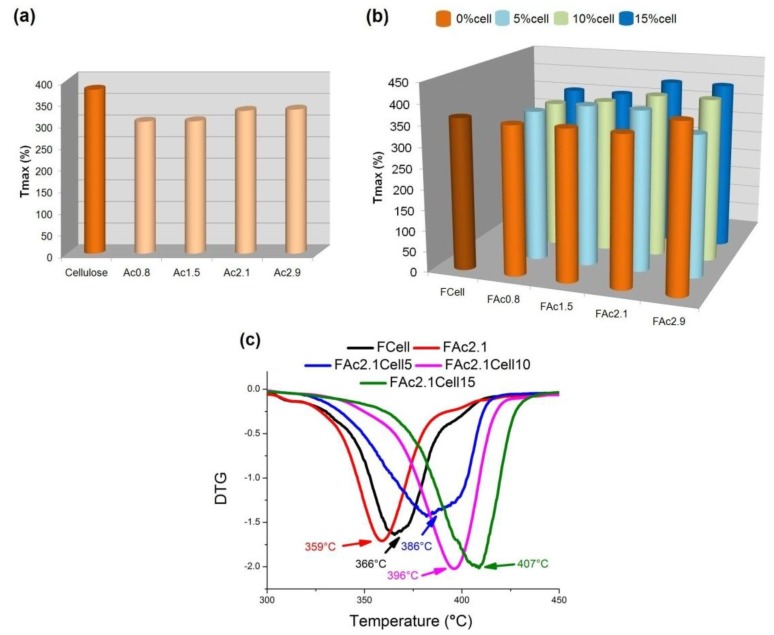
Temperature at the maximum degradation (T_max_) for (**a**) starting cellulose and cellulose acetate (AcDS); (**b**) cellulose (FCell), cellulose acetate (FAcDS) and bio-based cellulose acetate/cellulose (FAcDSCell%) films (**c**) DTG curves of FCell, FAc2.1, FAc2.1Cell5, FAc2.1Cell10 and FAC2.1Cell15.

The thermal stability of the films was investigated by TGA analysis and the temperature at which the rate of weight loss was at a maximum for these samples (T_max_) was detected according to the DTG curves (Figure 9b). The starting cellulose and cellulose acetates were also analyzed (Figure 9a). The DTG curves of FCell and all films prepared from FAc2.1 are shown in Figure 9c. The profiles exhibited by all other curves (not shown) were similar to these ones. 

The T_max_ of FCell (366 °C, Figure 9b) is lower than that of the starting cellulose (379 °C, Figure 9a). This decrease in T_max_ can be due to, at least partially, a decrease in the crystallinity from the starting cellulose to the prepared film. In the crystalline domains, the intermolecular hydrogen bonds between chains are stronger than those of non-crystalline regions and require more energy to break, as occurs in the step that precedes the thermal decomposition [48]. Additionally, powdered samples and their respective films have different surface areas, which may also have influenced the results.

The thermal decomposition of cellulose starts with dehydration and leads to anhydrocellulose. The hydroxyl group linked to C6 (C6OH) is involved in the reaction that leads to the formation of levoglucosan. C2OH and C3OH take part in the thermal decomposition reactions that result, e.g., in 1,2-anhydroglucose and 3-desoxyglucosanone, respectively ([48], and references therein).

When cellulose acetates are prepared, the reactivity of OH in the C6 position (C6OH) is favored compared to C2OH and C3OH because C6OH is the least sterically hindered group [56]. In a prior study, it was found that the order of reactivity was C6OH >> C2OH > C3OH. C3OH can still be involved in intramolecular hydrogen bonding and thus is less reactive than C2OH [46]. For Ac0.8 and Ac1.5, C6 is the most substituted when compared to C2 and C3. The T_max_ of these starting acetates were close and lower than the T_max_ of the starting cellulose (Figure 9a). This finding suggests that thermal decomposition involving C6COOCH_3_ requires less energy when compared to the levoglucosan formation from C6OH. The thermal decomposition of acetates involves the cleavage of the ester bond and the simultaneous removal of the acetyl group [48,57,58].

The T_max_ values of Ac2.1 and Ac2.9 are close and higher than the T_max_ of Ac0.8 and Ac1.5 (Figure 9a). As the total DS increases, C2 and C3 increase their individual DS. These results suggest that the thermal decomposition involving C2COOCH_3_ and C3COOCH_3_ requires more energy compared to C6COOCH_3_, in agreement with the results of a prior study on linter cellulose acetates [48]. Sisal cellulose acetates presented a similar behavior, *i.e*., T_max_ was shifted to higher temperature when DS increased [17]. The results of thermal analysis show that the stability of acetates with different DS seems to be dictated by the partial degree of substitution of C6, C2 and C3.

The films prepared from neat acetates presented a higher T_max_ than the respective starting acetates (Figure 9), despite their lower crystallinity, when compared to the powdered acetate (Figure 7). The organization of the chains in the films of acetates most likely led to strong intermolecular interactions compared to the powdered samples, which may have shifted the T_max_ to higher temperatures. As mentioned earlier, the differences in the surface areas of powdered samples and their respective films may also have influenced the results. No clear correlation was observed between DS and T_max_ for the films. A tendency to increase T_max_ when cellulose is present can be observed but with no clear correlation with the percentage of cellulose.

#### 2.3.7. Tensile Properties 

**Figure 10:**
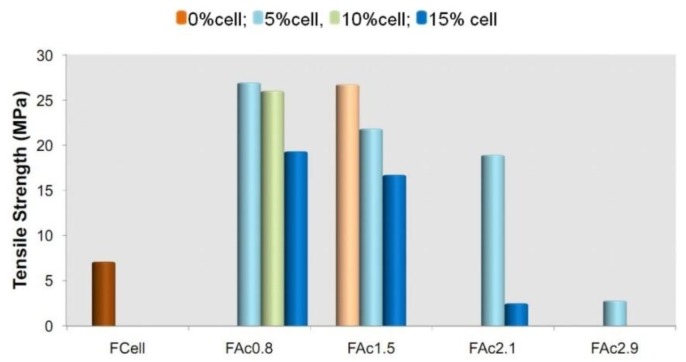
Tensile strength of cellulose (FCell), cellulose acetate (FAcDS) and bio-based cellulose acetate/cellulose (FAcDSCell%) films. The standard deviation ranging from 0% (FAc0.8Cell5) to 1.7% (FAc1.5Cell5)

In order to complete the exploration of the properties of the films based on linter cellulose and their acetates, the tensile strength was evaluated. In the present study, the fragility of some films prevented their analysis. Figure 10 shows the results for the films that were analyzed. The films were not exposed to humidity, as is often done, before assessing their mechanical properties. Absorbed moisture has a plasticizing effect and can impact the tensile properties. The elongation at break usually increases with relative humidity, as observed for bio-based films prepared from starch [59], methyl cellulose [60], chitosan [61], and myofibrilar protein [62]. The tensile strength quite often decreases with relative humidity [59], but in some studies, a positive effect was observed for this property, depending on the relative humidities employed [60,62,63] and also on other parameters, such as temperature [61].

Except for FAc2.1Cell and FAc2.9Cell5, all other films prepared exhibited tensile strength in the same range or higher than that observed for films based on hydroxypropylcellulose and microcrystalline cellulose fibers, where values from approximately 5 MPa (0% cellulose) to 11 MPa (20% cellulose) were found [64]. 

The cellulose film (FCell) presented a low tensile strength (Figure 10), suggesting that the micron-sized fibrous elements that were observed (Figure 4) may have behaved as regions of discontinuity of the material and decreased the tensile strength. 

The film prepared from the neat acetate DS 0.8 (FAc0.8) proved fragile and was not analyzed. However, when cellulose was mixed with the acetate, the films (FAc0.8Cell5, FAc0.8Cell10 and FAc0.8Cell15) could be analyzed and generated the best set of results (Figure 10). This indicates that domains composed of cellulose chains may have acted as a reinforcement of the acetate matrix. However, when the percentage of the cellulose present was increased (and FAc0.8Cell10, FAc0.8Cell15), a trend towards decreasing tensile strength was observed. As previously mentioned, the short linter cellulose chains have a high tendency to aggregate in LiCl/DMAc. When the concentration of cellulose increases, the formation of these aggregates is favored, and, in the preparation of films during the slow removal of the solvent, the formation of fibrous elements is also favored. This may have led to elements with dimensions in a range that decreased their strengthening action. 

The film prepared from neat cellulose acetate DS 1.5 (FAc1.5) showed good tensile strength when compared to the whole set of films, but the tensile strength decreased when cellulose was present (FAc1.5Cell5, FAc1.5Cell15) (Figure 10). This result suggested that the organization of cellulose chains favors the formation of the fibrous elements on a scale that makes them a factor for material discontinuity for the acetate with this DS.

The results of the tensile strength of some films prepared from sisal acetates mixed with sisal cellulose (previous study) indicated the reinforcement effect of the sisal cellulose. However, the tensile strengths of these films [17] were almost twice that observed in the present study**.** The longer sisal cellulose chains have a lower tendency to aggregate in LiCl/DMAc [26], which makes the formation of micron-sized fibrous elements difficult, unlike that which occurred with the films prepared from linter acetates mixed with linter cellulose (Figure 4 and Figure 5). The films prepared from sisal cellulose acetate plus cellulose exhibited good mechanical properties and optical transparency, suggesting that the strengthening of cellulose occurred at the nanoscale [17].

The films prepared from acetates with DS 2.1 and 2.9 (FAc2.1 and FAc2.9) exhibited low values of tensile strength (Figure 10). In this case, the microspheres observed in these films (Figure 4) may have acted as regions of discontinuity. Films prepared from sisal acetates, with DS in this interval, which also presented microspheres, exhibited results similar to the ones described here [17]. Nevertheless, in the present study, the presence of cellulose in the films at least enabled the analysis because the films prepared from the neat acetates were too fragile for this test.

The above results indicated that, depending on the dimensions of the fibrous elements present, they can impact positively or negatively on the tensile properties, although the polarity of the acetyl and hydroxyl groups fosters the interactions between acetates and the cellulose chain (Figure 1) at the interfaces of the matrix-fibrous elements [65]. It should be emphasized that in thin thickness composites, such as films, fibrous elements in nanoscale can act as reinforcement, while those in microscale can decrease the mechanical properties of these materials [66].

All of the films analyzed presented a very low percentage of elongation (between 0.60 ± 0.10, Fac2.9Cell15, and 1.60 ± 0.20, FAc0.8Cell15), which characterizes these films as rigid. This might have been caused by their crystallinity because overall they presented high crystallinity (Figure 7b). Regarding the tensile modulus, except for FAc2.1Cell15 and FAc2.9Cell5 (approximately 250 MPa ± 6% for both), all other films exhibited higher modulus than FCell (510 MPa ± 6.5%). The films based on acetates presented tensile modulus ranging from 1285 MPa ± 15% (FAc1.5) to 3420 MPa ± 7% (FAc2.1Cell5), and no clear correlation with DS and/or cellulose content was observed. The films prepared in the present study exhibited tensile modulus higher than films based on hydroxypropylcellulose and microcrystalline cellulose fibers, where tensile moduli ranged from approximately 100 MPa (0% cellulose) to 315 MPa (20 wt % cellulose) [64].

## 3. Experimental Section 

### 3.1. Materials

The low DP cotton linter employed was kindly supplied by Industria Fibra S/A (Americana, Sao Paulo). DMAc (Synth) was purified by distillation with CaH_2_ under nitrogen followed by storage over 4 Å molecular sieves. Acetic anhydride (Ac_2_O, Mallinckrodt) was distilled from P_4_O_10_ prior to use. LiCl (Synth) was dried for 3 h at 200 °C and stored in a desiccator. Methanol (Synth), cupriethylenediamine (Qeel), sodium chloride (Synth), potassium carbonate (Synth) and sodium hydroxide (Synth) were used as received.

### 3.2. Cellulose Pre-Treatment

Cellulose mercerization was carried out as given elsewhere [46]. In a solution of 20% NaOH (1:50 w/w), linter cellulose was stirred for 1 h at 0 °C [46]. After this period, the slurry was washed with distilled water until the pH was equal to that of the starting distilled water. The mercerized linter cellulose was kept at room temperature for 24 h and then dried under reduced pressure at 60 °C until constant weight. 

The following procedures were performed for the mercerized linter cellulose, hereafter called only linter cellulose or cellulose.

### 3.3. Dissolution and Acetylation of Cellulose 

**Figure 11:**
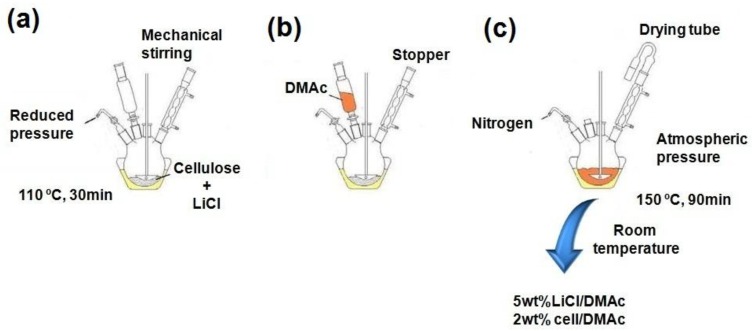
Schematic representation of the dissolution of cellulose.

Following the procedure established in a previous work [25,26], a four-necked round-bottomed flask (250 mL) was equipped with a stopcock, mechanical stirrer, condenser closed with a stopper and cylindrical funnel (without equilibration sidearm). A mixture of cellulose (2.0 g) and LiCl (5.0 g) were added into the flask that was connected to a vacuum pump and immersed in an oil bath, whose temperature was externally controlled (FE50RP controller, Flyever, São Carlos, Brazil). The pressure was reduced to 2 mm Hg, and the mixture was heated from room temperature to 110 °C and maintained for 30 min (Figure 11a). The vacuum pump was turned off and 100 mL of DMAc was slowly added (Figure 11b). The system was then brought to atmospheric pressure with dry oxygen-free nitrogen, and the condenser was provided with a drying tube (Figure 11c). The mixture was heated to 150 °C, and was vigorously stirred for 90 min. The reaction mixture was then cooled to room temperature and allowed to stir overnight.

The cellulose acetates were obtained by following a procedure described elsewhere [26]. 

### 3.4. Characterization of Cellulose Acetates

#### 3.4.1. Degree of Substitution (DS) 

**^1^**H NMR spectra of cellulose acetates were recorded on a Brucker AC-200 spectrometer operating at 200 MHz at 80 °C using 392 scans. The analysis was performed as described elsewhere [45,46,47,48].

### 3.5. Elaboration of the Films

#### 3.5.1. Cellulose Acetate Films

The cellulose acetates obtained with different DS were solubilized in LiCl/DMAc by following the experimental procedure already described for the dissolution of the linter cellulose, with some modifications. A mixture of cellulose acetate (2.5 g) and LiCl (2.5 g) was added to the flask. The system was heated to 110 °C for 1 h under a vacuum pump and a mechanical stirrer. After this period, 50.0 mL of distilled DMAc was added into the flask, and the vacuum pump was turned off and replaced by a reflux condenser under a flow of N_2_. The mixture was heated to 160 °C under vigorous stirring for 1.5 h. To obtain homogeneous solution, the mixture was stirred for 24 h at room temperature. After this period, the solution was filtered through 47 mm disks of a glass microfiber filter paper (MGC grade) under positive pressure and then immediately cast on a glass Petri dish (diameter 5.8 cm), and left standing at room temperature overnight. During this time, a gel-like material was formed with a consistency suitable for washing. The materials were then exhaustively washed with distilled water. To check the elimination of the LiCl in this step, the conductivity of running water was determined by using a Schott Handylab LF1 conductometer, and afterwards by atomic absorption (see Section 3.6.3). The elimination of DMAc was checked by N elemental analysis (see 3.6.3.). Importantly, DMAc can be efficiently recovered from water [67].

The residual water was eliminated by drying the formed films at room temperature for one day, and then at 60 °C under reduced pressure, until a constant weight. In these final steps, the films were put between two Teflon^®^ plates to avoid their furling.

#### 3.5.2. Cellulose Film

The cellulose film was obtained by mixing 2.5 g cellulose and 2.5 g LiCl in 50.0 mL distilled DMAc. The cellulose film was prepared in the LiCl/DMAc solution following the same steps mentioned for cellulose acetate films.

#### 3.5.3. Cellulose Acetate/Cellulose Films

Bio-based films based on cellulose acetates and cellulose were prepared using LiCl/DMAc as the solvent system. Cellulose acetates with DS values of 0.8, 1.5, 2.1 and 2.9 and different amounts of cellulose (5, 10 and 15 wt %) were considered. The bio-based cellulose acetate/cellulose films were prepared in the LiCl/DMAc solution following the same steps as mentioned for the cellulose acetate films.

### 3.6. Characterization of Starting Materials and Cellulose Acetate/Cellulose Films

#### 3.6.1. Crystallinity Index (Ic) 

X-ray diffraction analysis was employed to evaluate the crystallinity index (Ic) of cellulose, using a VEB CARL ZEISS-JENA URD-6 Universal Diffractometer operating at 40 kV/20 mA and k (Cu Ka) = 1.5406 Å. Crystallinity index (Ic) of cellulose acetates and cellulose acetate/cellulose film were evaluated as described in previous papers [25,46,47]. 

#### 3.6.2. Thermogravimetric Analysis (TGA)

TGA analyses were conducted in a Shimadzu TGA-50 instrument for samples of cellulose, cellulose acetates and bio-based cellulose acetate/cellulose films, as described elsewhere [48].

#### 3.6.3. Elemental Analysis and Atomic Absorption

The films were characterized by elemental nitrogen analysis (Perkin Elmer, Elemental Analysis 2400) and atomic absorption lithium analysis (Hitachi, Z-8100). The original samples of cellulose and cellulose acetates were also analyzed. 

#### 3.6.4. Determination of Molar Mass Distribution by Size-Exclusion Chromatography (SEC)

The molar mass distribution of acetate DS 0.8 before and after film preparation and with or without cellulose was determined by size-exclusion chromatography. The analysis was performed at 60 °C using a Shimadzu SCL-10A system controller connected to a LC-10AD with a refractive index detector (RID-6A) and a CTO 10 column oven. The pre-column system consisted of a 10-µm mixed PLgel. The mobile phase was DMAc/0.5% LiCl, flowing at 0.6 mL min^−1^ and at a pressure of 41 kgf cm^−2^. The column calibration was based on a set of pullulan standards with molar masses of 1.60 × 10^6^, 3.80 × 10^5^, 2.12 × 10^5^, 1.00 × 10^5^, 4.80 × 10^4^, 2.37 × 10^4^, 1.22 × 10^4^, 5.80 × 10^3^, 738 and 180 g mol^−1^. 

The sample concentration was 2 mg mL^−1^ LiCl/DMAc. The samples were solubilized using the same conditions for the dissolution of the linter cellulose. The system was heated to 160 °C under vigorous stirring for 1.5 h in a glass reactor equipped with a mechanical stirrer and a reflux condenser under nitrogen flow. After this period, the mixture was cooled to room temperature and allowed to stir overnight. Before being injected into the SEC system, the solutions were filtered through a 1.2-µm Glassfase Mikrofilter-GMF 3 membrane filter. 

#### 3.6.5. Morphological Analyses

##### ***Scanning Electron Microscopy (SEM)*** 

The films were examined by Scanning Electron Microscopy (SEM) using a LEO 440 ZEISS/LEICA model as described elsewhere [30].

##### ***Atomic Force Microscopy (AFM)*** 

Atomic Force Microscopy (AFM) measurements were performed for some films in a Dimension V (VEECO Co.) microscope in the intermittent contact mode in air at room temperature using silicon cantilevers with a resonance frequency close to 190 kHz. The image size was 5 × 5 µm² with a resolution of 512 × 512 pixels. The images were only height-corrected after measurements. Image processing and the determination of the root-mean-square (RMS) were performed by using the Gwyddion software of the AFM microscope.

#### 3.6.6. Humidity Absorption 

The film samples were stored in airtight glass jars containing saturated salt solutions to give different relative humidities, and low, medium and high humidity (12%, 43% and 75%, respectively) were considered. The set of salt solutions and relative humidities (RH) used in this experiment were LiCl (RH = 12%), K_2_CO_3_ (RH = 43%) and NaCl (RH = 75%) [68]. 

Before the analysis, the films were dried at 105 °C for 24 h. The tests were conducted using approximately 0.20 g of the film deposited on aluminum dishes. The samples were conditioned for a period of 30 days to ensure equilibrium. 

#### 3.6.7. Tensile Tests 

The tensile tests were performed in a TA Instruments model 2980 operating in tension film mode (23 ± 1 °C). The film samples were strained at a constant rate of 1 N min^−1^ until 18 N or failure. Samples 6.3 mm in width and 10 mm in gauge length were used for the tests. At least five specimens of each type of film were used for the test. 

## 4. Conclusions 

The AFM analysis showed that, in general, the presence of cellulose increased both the asperity thickness and the surface roughness of the analyzed films, indicating that cellulose chains are at least partially organized in domains and not molecularly dispersed within acetate chains. The films prepared from solutions of cellulose acetates mixed with cellulose (5, 10 and 15 wt %) exhibited fibrous structures on their surfaces. These structures probably were formed through aggregation of cellulose chains into domains, due to strong intermolecular interactions. Overall, the presence of cellulose led to films with less affinity for water, also suggesting that the cellulose chains auto-organized into domains, as indicated by other properties. The thermal stability of all films considered in this study showed no substantial difference.

The results of this study underline the need to explore the influence of properties of different starting celluloses, such as the average molar mass and the crystallinity, among others, when they are subjected to the same process. The films prepared with linter as a starting material presented more differences than similarities when compared to films prepared from sisal cellulose, although the linter and sisal acetates, and their respective films, were prepared using the same procedure.

The rigidity of the linter films, together with the presence of microspheres in some of them have potential use, in principle, as scaffold coatings in tissue engineering and/or drug delivery.

## References

[1] Silva C.G., Grelier S., Pichavant F., Frollini E., Castellan A. (2013). Adding value to lignins isolated from sugarcane bagasse and Miscanthus. Ind. Crop. Prod..

[2] Ramires E.C., Frollini E. (2012). Tannin phenolic resins: Synthesis, characterization, and application as matrix in biobased composites reinforced with sisal fibers. Compos. Part B Eng..

[3] Castro D.O., Ruvolo-Filho A., Frollini E. (2012). Materials prepared from biopolyethylene and curaua fibers: Composites from biomass. Polym. Test..

[4] Silva C.G., Oliveira F., Ramires E.C., Castellan A., Frollini E. (2012). Composites from a forest biorefinery by-product and agrofibers: Lignosulfonate-Phenolic type matrices reinforced with sisal fibers. Tappi J..

[5] Cerrutti B.M., Souza C.S., Castellan A., Ruggiero R., Frollini E. (2012). Carboxymethyl lignin as stabilizing agent in aqueous ceramic suspensions. Ind. Crop. Prod..

[6] Cao X., Sun S., Peng X., Zhong L., Sun R. (2013). Synthesis and characterization of cyanoethyl hemicelluloses and their hydrated products. Cellulose.

[7] Bujanovic B., Ralph S., Reiner R., Hirth K., Atalla R. (2010). Polyoxometalates in oxidative delignification of chemical pulps: Effect on lignin. Materials.

[8] Pinto R.J.B., Neves M.C., Pascoal Neto C., Tito Trindade T. (2012). Growth and chemical stability of copper nanostructures on cellulosic fibers. Eur. J. Inorg. Chem..

[9] Foston M.B., Hubbell C.A., Ragauskas A.J. (2011). Cellulose isolation methodology for NMR analysis of cellulose ultrastructure. Materials.

[10] De Paula M.P., Lacerda T.M., Zambon M.D., Frollini E. (2012). Adding value to the Brazilian sisal: Acid hydrolysis of its pulp seeking production of sugars and materials. Cellulose.

[11] Lacerda T.M., de Paula M.P., Zambon M.D., Frollini E. (2012). Saccharification of Brazilian sisal pulp: Evaluating the impact of mercerization on non-hydrolyzed pulp and hydrolysis products. Cellulose.

[12] Lacerda T.M., Zambon M.D., Frollini E. (2013). Effect of acid concentration and pulp properties on hydrolysis reactions of mercerized sisal. Carbohydr. Polym..

[13] Kaur U., Oberoi H.S., Bhargav V.K., Sharma-Shivappa R., Dhaliwal S.S. (2012). Ethanol production rom alkali- and ozone-treated cotton stalks using thermotolerant Pichia kudriavzevii HOP-1. Ind. Crop. Prod..

[14] Dutta S., De S., Alam M.I., Abu-Omar M.M., Saha B. (2012). Direct conversion of cellulose and lignocellulosic biomass into chemicals and biofuel with metal chloride catalysts. J. Catal..

[15] Mazzoli R., Lamberti C., Pessione E. (2011). Engineering new metabolic capabilities in bacteria: Lessons from recombinant cellulolytic strategies. Trends Biotechnol..

[16] Fernandes S.C.M., Freire C.S.R., Silvestre A.J.D., Pascoal Neto C., Gandini A. (2011). Novel materials based on chitosan and cellulose. Polym. Int..

[17] Almeida E.V.R., Morgado D.L., Ramos L.A., Frollini E. (2013). Sisal cellulose and its acetates: Generation of films and reinforcement in a one-pot process. Cellulose.

[18] Yang Q., Saito T., Isogai A. (2012). Facile fabrication of transparent cellulose films with high water repellency and gas barrier properties. Cellulose.

[19] Trovatti E., Fernandes S.C.M., Rubatat L., da Silva Perez D., Freire C.S.R., Silvestre A.J.D., Pascoal Neto C. (2012). Pullulan–nanofibrillated cellulose composite films with improved thermal and mechanical properties. Compos. Sci. Technol..

[20] Peydecastaing J., Vaca-Garcia C., Borredon E. (2011). Bi-acylation of cellulose: Determining the relative reactivities of the acetyl and fatty-acyl moieties. Cellulose.

[21] Athauda T.J., Ozer R.R. (2012). Investigation of the effect of dual-size coatings on the hydrophobicity of cotton surface. Cellulose.

[22] Budimir A., Vukusic S.B., Flincec S.G. (2012). Study of antimicrobial properties of cotton medical textiles treated with citric acid and dried/cured by microwaves. Cellulose.

[23] Cardoso G.D., Alves P.L.C.A., Severino L.S., Vale L.S. (2011). Critical periods of weed control in naturally green colored cotton BRS Verde. Ind. Crop. Prod..

[24] Nourbakhsh S., Ashjaran A. (2012). Laser treatment of cotton fabric for durable antibacterial properties of silver nanoparticles. Materials.

[25] Ramos L.A., Assaf J.M., El Seoud O.A., Frollini E. (2005). Influence of supra-molecular structure and physico-chemical properties on the dissolution of celluloses in lithium chloride/*N*,*N*-dimethylacetamide solvent system. Biomacromolecules.

[26] Ramos L.A., Morgado D.L., El Seoud O.A., da Silva V.C., Frollini E. (2011). Acetylation of cellulose in LiCl-*N*,*N*-dimethylacetamie: First report on the correlation between the reaction efficiency and the aggregation number of dissolved cellulose. Cellulose.

[27] Ramos L.A., Morgado D.L., Gessner F., Frollini E., El Seoud O.A. (2011). A physical organic chemistry approach to dissolution of cellulose: Effects of cellulose mercerization on its properties and on the kinetics of its decrystallization. Arkivoc.

[28] De Paula M.P., Lacerda T.M., Frollini E. (2008). Sisal cellulose acetates obtaines from heterogeneous reactions. Express Polym. Lett..

[29] Almeida E.V.R., Frollini E., Castellan A., Coma V. (2010). Chitosan, sisal cellulose, and biocomposite chitosan/sisal cellulose films prepared from thiourea/NaOH aqueous solution. Carbohydr. Polym..

[30] Morgado D.L., Frollini E., Castellan A., Rosa D.S., Coma V. (2011). Biobased films prepared from NaOH/thiourea aqueous solution of chitosan and linter cellulose. Cellulose.

[31] Gericke M., Fardim P., Heinze T. (2012). Ionic liquids—Promising but challenging solvents for homogeneous derivatization of cellulose. Molecules.

[32] El Seoud O.A., Koschella A., Fidale L.C., Dorn S., Heinze T. (2007). Applications of ionic liquids in carbohydrate chemistry: A window of opportunities. Biomacromolecules.

[33] Sathitsuksanoh N., Zhu Z., Zhang Y.-H.P. (2012). Cellulose solvent-based pretreatment for corn stover and avicel: Concentrated phosphoric acid versus ionic liquid [BMIM]Cl. Cellulose.

[34] Nawaz H., Casarano R., El Seoud O.A. (2012). First report on the kinetics of the uncatalyzed esterification of cellulose under homogeneous reaction conditions: A rationale for the effect of carboxylic acid anhydride chain-length on the degree of biopolymer substitution. Cellulose.

[35] Glasser W.G., Becker U., Todd J.G. (2000). Novel cellulose derivatives. Part VI. Preparation and thermal analysis of two novel cellulose esters with fluorine-containing substituents. Carbohydr. Polym..

[36] Raus V., Sturcova A., Dybal J., Slouf M., Vackova T., Salek P., Kobera L., Vlcek P. (2012). Activation of cellulose by 1,4-dioxane for dissolution in *N*,*N*-dimethylacetamide/LiCl. Cellulose.

[37] Crepy L., Monchau F., Chai F., Raoul G., Hivart P., Hildebrand H.F., Martin P., Joly N. (2012). Evaluation of a bio-based hydrophobic cellulose laurate film as biomaterial—Study on biodegradation and cytocompatibility. J. Biomed. Mater. Res. B.

[38] Liu H., Kar N., Edgar K.J. (2012). Direct synthesis of cellulose adipate derivatives using adipic anhydride. Cellulose.

[39] Regiani A.M., Frollini E., Marson G.A., Arantes G.M., El Seoud O.A. (1999). Some aspects of acylation of cellulose under homogeneous solution conditions. J. Polym. Sci. A Polym. Chem..

[40] Chen W., Su Y., Zheng L., Wang L., Jiang Z. (2009). The improvement of oil/water separation perfomance of cellulose acetate-graft-polyacrylonitrile membranes. J. Membr. Sci..

[41] Ho F.F.L., Klosiewicz D.W. (2002). *Proton nuclear* magnetic resonance spectrometry for determination of substituents and their distribution in carboxymethylcellulose. Anal. Chem..

[42] Mohammadi T., Saljoughi E. (2009). Effect of production conditions on morphology and permeability of asymmetric cellulose acetate membranes. Desalination.

[43] Amim J., Petri D.F.S., Maia F.C.B., Miranda P.B. (2010). Ultrafhin cellulose ester films: Preparation, characterization and protein immobilization. Quim. Nova.

[44] Morgado D.L., Martins V.C.A., Plepis A.M.G., Frollini E. (2011). Aggregation of cellulose acetates chains in LiCl/DMAc: Evaluation via viscometry. Polímeros.

[45] Edgar K.J., Arnold K.M., Blount W.W., Lawniczak J.E., Lowman D.W. (1995). Synthesis and properties of cellulose acetates. Macromolecules.

[46] Ass B.A.P., Belgacem M.N., Frollini E. (2006). Mercerized linter cellulose: Characterization and acetylation in *N*,*N*-dimethylacetamide/lithium chloride. Carbohydr. Polym..

[47] Ciacco G.T., Morgado D.L., Frollini E., Possidonio S., El Seoud O.A. (2010). Some aspects of acetylation of untreated and mercerized sisal cellulose. J. Braz. Chem. Soc..

[48] Morgado D.L., Frollini E. (2011). Thermal decomposition of mercerized linter cellulose and its acetates obtained from a homogeneous reaction. Polímeros.

[49] Dort I. (1988). On the possibility of quantitative evaluation of polymer-solvent interaction from the Huggins viscosity constant. Polymer.

[50] McCormick C.L., Nonaka T., Johnson C.B. (1988). Water-soluble copolymers: 27 Synthesis and aqueous solution behavior of associative acrylamide/*N*-alkylacrylamide copolymers. Polymer.

[51] Roy C., Budtova T., Navard P. (2003). Rheological properties and gelation of aqueous cellulose-NaOH solutions. Biomacromolecules.

[52] El Seoud O.A., Marson G.A., Ciacco G.T., Frollini E. (2000). An efficient, one-pot acylation of cellulose under homogeneous reaction conditions. Macromol. Chem. Physic..

[53] Zhou H., Chen X. (2008). Characteristics and degradation of chitosan/cellulose acetate microspheres with different model drugs. Front. Mater. Sci. China.

[54] Wang F., Yang Y., Zhang X., Zhu X., Chung T., Moochhala S. (2002). Cellulose acetate membranes for transdermal delivery of scopolamine base. Mater. Sci. Eng. C.

[55] Meier M., Kanis L.A., Soldi V. (2004). Characterization and drug-permeation profiles of microporous and dense cellulose acetate membranes: influence of plasticizer and pore forming agent. Int. J. Pharmaceut..

[56] Marson G.A., El Seoud O.A. (1999). A novel, efficient procedure for acylation of cellulose under homogeneous solution conditions. J. Appl. Polym. Sci..

[57] Huang M.-R., Li X.-G. (1998). Thermal degradation of cellulose and cellulose esters. J. Appl. Polym. Sci..

[58] Li X.G. (1999). High-Resolution thermogravimetry of cellulose esters. J. Appl. Polym. Sci..

[59] Lawton J.W. (1996). Effect of starch type on the properties of starch containing films. Carbohydr. Polym..

[60] Rachtanapun P., Wongchaiya P. (2012). Effect of relative humidity on mechanical properties of blended chitosan methylcellulose film. Chiang Mai J. Sci..

[61] Srinivasa P.C., Ravi R., Tharanathan R.N. (2007). Effect of storage conditions on the tensile properties of eco-friendly chitosan films by response surface methodology. J. Food Eng..

[62] Cuq B., Gontard N., Aymard C., Guilbert S. (1997). Relative humidity and temperature effects on mechanical and water vapor barrier properties of myofibrillar protein-based films. Polym. Gels Netw..

[63] Chang Y.P., Cheah P.B., Seow C.C. (2000). Plasticizing-antiplasticizing effects of water on physical properties of tapioca starch films in the glassy state. J. Food Sci..

[64] Borges J.P., Godinho M.H., Martins A.F., Stamatialis D.F., de Pinho M.N., Belgacem M.N. (2004). Tensile properties of cellulose fiber reinforced hydroxypropylcellulose films. Polym. Compos..

[65] Mohanty A.K., Wibowo A., Misra M., Drzal L.T. (2004). Effect of process engineering on the performance of natural fiber reinforced cellulose acetate biocomposites. Compos. A Appl. Sci. Manuf..

[66] Panaitescu D.M., Frone A.M., Ghiurea M., Spataru C.I., Radovici C., Iorga M.D. (2001). Properties of Polymer Composites with Cellulose Microfibrils. Advances in Composite Materials—Ecodesign and Analysis.

[67] Cheremisinoff N.P. (2000). Evaporating and Drying Equipment. Handbook of Chemical Processing Equipment.

[68] American Society for Testing and Materials (ASTM) (2003). Standard Practice for Maintaining Constant Relative Humidity by Means of Aqueous Solutions, ASTM E 104–02. Annual Book of ASTM Standard.

